# Real-world impact of intrathecal baclofen therapy: the benefits of neteka on patient outcomes, caregiver burden, and cost efficiency

**DOI:** 10.1007/s10072-024-07965-z

**Published:** 2025-01-13

**Authors:** Manuela D’Ercole, Benedetta Burattini, Quintino Giorgio D’Alessandris, Maria Filomena Fuggetta, Francesco Pambianco, Francesca Annunziata, Alessandro Izzo, Nicola Montano

**Affiliations:** 1 Department of Neurosurgery, Fondazione Policlinico Universitario A. Gemelli IRCCS, Largo A. Gemelli 8, Rome, 00168 Italy; 2 Department of Neuroscience, Università Cattolica del Sacro Cuore, Largo F. Vito, 1, Rome, 00168 Italy

**Keywords:** Intrathecal baclofen therapy, Half-life, Pump refill, Quality of life, Healthcare cost

## Abstract

**Background:**

Intrathecal baclofen therapy (ITB) is a well-established treatment modality for severe spasticity, but it is burdened by the need for periodic pump refills. The introduction of a new formulation of baclofen with an extended stability of 180 days (Neteka, Nordic Group BV) could decrease the frequency of refills. We aimed at analyzing the clinical and economic impact of Neteka introduction in our outpatient facility.

**Methods:**

Forty-eight patients with an implanted device for intrathecal baclofen infusion, who shifted from standard baclofen to Neteka, were included in the present study. We collected clinical and refilling data both from pre-Neteka and Neteka timeframes.

**Results:**

In patients treated with baclofen 2 mg/ml (45/48, 93.8%), the introduction of Neteka led to a significant decrease of median number of refills per year (2.7 vs. 4 in pre-Neteka timeframe, *p* < 0.0001). This resulted in an improved quality of life, as shown by the reduction of Oberst Caregiving Burden Scale score both for time and difficulty of caregiving (*p* < 0.0001 and *p* = 0.0003, respectively). Furthermore, a median saving of 302.40 euro per year per patient, as compared to pre-Neteka timeframe, was registered. Similar results were obtained in the smaller cohort treated with baclofen 0.5 mg/ml (3/48, 6.3%). Neteka appreciation rate was high. As expected, no changes in clinical effectiveness of ITB therapy were observed by comparing pre-Neteka and Neteka timeframes.

**Conclusions:**

The extended half-life provided by Neteka, by reducing the number of refills, could improve patient quality of life, enhance caregiver satisfaction, and decrease the financial burden on healthcare system.

## Introduction

Intrathecal baclofen therapy (ITB) is a well-established treatment modality for managing severe spasticity in patients with neurological conditions such as multiple sclerosis, spinal cord injuries, and cerebral palsy [1–3]. The administration of baclofen via an intrathecal pump allows for the delivery of the drug directly to the cerebrospinal fluid, providing effective spasticity control with lower systemic doses compared to oral administration [1].

These conditions significantly affect the patient’s quality of life, as well as impose substantial challenges on caregivers and financial burdens on the healthcare system [4]. One of the main challenges associated with ITB is the need for periodic pump refills [5], which typically require hospital visits and can be burdensome for patients and caregivers.

For many years, the drugs provided by Novartis Farma and Bioindustria L.I.M. have been used, which are highly effective, but their pharmacokinetic profile necessitates relatively frequent pump refills, typically every few months, depending on the patient’s dosage requirements. The introduction of a new baclofen brand, Neteka, promises to address this issue by offering an extended stability, potentially allowing for longer intervals between refills without compromising therapeutic efficacy.

This study aims to evaluate the practical benefits of Neteka in a clinical setting, focusing on the reduction of refill frequency, associated cost savings, and the impact on caregiver burden.

## Methods

### Patients enrollment

Patients suffering from spastic tetraparesis or paraparesis, with an implanted device for intrathecal infusion of baclofen (Synchromed II, Medtronic, Minneapolis, MN, USA) and followed at the Functional Neurosurgery Division of our Institution were included in the present study. These patients undergo periodical outpatient reservoir refilling with 20 ml baclofen solution. The study was conducted according to the principles set forth in 1964 Declaration of Helsinki and later amendments. Patients signed an informed consent form according to the scientific purposes of the Institutional Ethics Committee.

### Baclofen pharmacological products

Baclofen vials for intrathecal administration exist in two different concentrations: 2 mg/ml and 0.5 mg/ml (Table 1). The concentration which is used for reservoir refilling depends on ITB dosage. Three different baclofen brands are in use at our institution: Lioresal (Novartis Farma, Varese, Italy), Baclofene Bioindustria L.I.M. (Bioindustria L.I.M., Novi Ligure, Italy), Neteka (Nordic Group BV, Hoofddorp, The Netherlands) (Table 1). Lioresal and Baclofene Bioindustria L.I.M. have a guaranteed stability in the intrathecal infusion system of 11 weeks (77 days), while Neteka has a guaranteed stability in the intrathecal infusion system of 180 days. As concerns official costs, one vial of Lioresal 2 mg/ml 5 ml costs 120.72€, one vial of Baclofene Bioindustria L.I.M. 0.5 mg/ml 20 ml costs 73.24€, one vial of Baclofene Bioindustria L.I.M. 2 mg/ml 5 ml costs 66.10€, one vial of Neteka 0.5 mg/ml 20 ml costs 66.11€ and one vial of Neteka 2 mg/ml 5 ml costs 62.80 euro. Neteka is available also as 2 mg/ml 20 ml single vial, whose cost perfectly equals the cost of four 5 ml vials (Table 1). No patient at our Institution is treated with Lioresal 0.5 mg/ml.

**Table 1:** Baclofen formulations available at our Institution

Brand name	Manufacturer	Drug concentration
Lioresal	Novartis Farma	10 mg/20 ml 10 mg/5 ml
Baclofene Bioindustria L.I.M.	Bioindustria L.I.M.	10 mg/20 ml 10 mg/5 ml
Neteka	Nordic Group BV	10 mg/20 ml 10 mg/5 ml 40 mg/20 ml

### Retrieval of clinical data

Clinical data and refilling details are prospectively collected as part of routine clinical management. Starting from November 2022, we have gradually introduced Neteka in our outpatient facility, due to its long-lasting stability. Therefore, from our prospectively collected database, we collected refilling data in the 1-year timeframes May 2021-April 2022 (timeframe 1, before introduction of Neteka) and May 2023-April 2024 (timeframe 2, after the completion of Neteka introduction). We also collected spasticity grade, measured as mean Ashworth value in the four limbs, both pre and post Neteka introduction. Further, we administered a dedicated questionnaire evaluating the time spent and difficulties encountered for patients caregiving, the Oberst Caregiving Burden Scale (OCBS) [6], both before and after Neteka introduction. The OCBS questionnaire is a two-dimensional 15-item scale in which each item can be rated using a 5-point Likert scale from 1 (none) to a 5 (a great deal) with respect to both time and difficulty. Therefore, a total final score ranging from 15 (no time/difficulty) to 75 (maximum time/difficulty) can be recorded. Notably, the OCBS can be filed either by the patient or by the caregiver. Finally, we asked patients to rate their appreciation for Neteka introduction following a scale spanning from 1 (no appreciation) to 5 (maximum appreciation).

### Statistical analysis

Data were described as mean ± standard deviation or median and range, or interquartile range when appropriate. Comparison of continuous variables between the two timeframes was performed using the Wilcoxon test for paired samples. MedCalc ver 20.015 (MedCalc Software Ltd., Ostend, Belgium) was used for statistical analysis. A *p* < 0.05 was considered as significant.

## Results

### Patients characteristics

In the study period, 49 patients were followed at our outpatient facility for periodical reservoir refilling with baclofen. One patient was excluded because she could be treated only with Lioresal brand due to intolerance to other baclofen formulations. The 48 patients who shifted the baclofen brand from Baclofene Bioindustria L.I.M. to Neteka thus built our study group. There were 37 males and 11 females, with a mean age 44.3 ± 19.3 years. The majority of patients (45/48, 93.8%) were in treatment with baclofen 2 mg/ml, while 3 patients (6.3%) used baclofen 0.5 mg/ml. Mean daily dose of intrathecal baclofen was 347.1 ± 176.1 µg in the former and 52.0 ± 17.7 µg in the latter. No dose changes were registered in the study period.

### Number of refills and vials

Thirty-two patients (66.7%) experienced a reduction in the number of refills, and consequently of the outpatient visits, in timeframe 2 as compared to timeframe 1; the median number of visits reduction was 2 [range, 1–2].

Comparison of number of refills and vials per year and relative costs between timeframes 1 and 2 are shown in Table 2. Regarding patients treated with baclofen 2 mg/ml, we observed a significant reduction of the number of refills and total vials used per patient and, consequently, of the yearly cost of treatment (*p* < 0.0001 for all three variables, respectively; Wilcoxon test for paired samples). Overall, yearly baclofen cost for the 45 patients treated with the 2 mg/ml concentration was reduced by 14,128 € (from 53,064€ in timeframe 1 to 38,936 € in timeframe 2). As concerns the number of vials, since the 40 mg/20 ml vial is available only for Neteka, in order to allow a sensible comparison with Baclofene Bioindustria L.I.M., we considered it as equivalent to four 10 mg/5 ml vials, a formulation available for both brands (Table 1). Regarding the 3 patients treated with baclofen 0.5 mg/ml, we observed a reduction in the number of yearly refills and vials used, and consequently of the costs, also in this group. However, formal statistical analysis could not be performed due to the low numerosity. The yearly savings for these 3 patients were 418.03 € overall.

**Table 2:** Comparison of number of refills, vials and relative costs between timeframe 1 and 2

	Baclofen 2 mg/ml	Baclofen 0.5 mg/ml
*N*	45	3
*n* refills/year		
timeframe 1	4 [4–5]	4 [4–4]
timeframe 2	2.7 [1.9–4.3]	2.0 [1.2–2.5]
p*	< 0.0001	NA
*n* vials/year^#^		
timeframe 1	16 [16–20]	4 [4–4]
timeframe 2	12 [8–20]	2 [2–3]
p*	< 0.0001	NA
Treatment cost per year (€)		
timeframe 1	1056 [1056–1320]	293.60 [293.60-293.60]
timeframe 2	753.60 [502.40–1256.00]	132.22 [132.22-198.33]
p*	< 0.0001	NA

*Wilcoxon test; NA (not applicable) results are due to low numerosity. ^#^5 ml 2 mg/ml and 20 ml 0.5 mg/ml vials for both brands were compared

To better identify the subgroup of patients who most benefit from Neteka introduction, we calculated the theoretical interval between two consecutive refills, dividing the total amount of Baclofen contained in 20 ml of solution by the daily baclofen dose. We then divided patients in two groups, those with an interval ≤ 77 days and those with an interval > 77 days, with 77 days corresponding to the guaranteed stability time of Lioresal and Baclofene Bioindustria L.I.M. Table 3 details the comparison of number of refills, vials and costs per year between timeframes 1 and 2, considering patients with interval between refills ≤ 77 days or > 77 days. Results are limited to patients treated with baclofen 2 ng/ml, since all patients treated with baclofen 0.5 mg/ml have an interval > 77 days. As expected, the benefit derived from the introduction of Neteka was particularly pronounced in patients with an interval between refills > 77 days. For these patients, in fact, the introduction of Neteka allowed to avoid early refills due to loss of stability of the drug, thus approaching the actual interval between refills to the theoretical one.

**Table 3:** Comparison of number of refills, vials and relative costs between timeframe 1 and 2 depending on interval between refills (≤ 77 days vs. > 77 days) in patients treated with baclofen 2 mg/ml

	Interval ≤ 77 days	Interva, > 77 days
*n*	9	36
*n* refills/year		
timeframe 1	6 [5.8–6.3]	4 [4–4]
timeframe 2	5.6 [5.3–6.1]	2.4 [1.6–3.2]
p*	0.0391	< 0.0001
*n* vials/year^#^		
timeframe 1	24 [23–25]	16 [16–16]
timeframe 2	24 [23–25]	12 [8–12]
p*	> 0.99	< 0.0001
Treatment cost per year (€)		
timeframe 1	1584 [1518–1650]	1056 [1056–1056]
timeframe 2	1507.20 [1444.40–1570.00]	753.60 [502.40-753.60]
p*	< 0.0001	< 0.0001

### Quality of life

The reduction in the number of refills and, consequently, of the outpatient visits, positively affected the quality of life of patients (Table 4). In the baclofen 2 mg/ml group, we observed a significant reduction in the OCBS score both for time and difficulty (*p* < 0.0001 and *p* = 0.0003, respectively; Wilcoxon test for paired samples), suggesting an improved quality of life. An OCBS score reduction was registered also in patients treated with baclofen 0.5 mg/ml, but again the numerosity was too low for formal statistical comparison. As a result, appreciation rate for Neteka was very high in both groups (Table 4). Of note, the vast majority of patients (45/48, 93.8%) had to arrange transportation to our outpatient facility by their own, with 11 of them (22.9%) forced to use an ambulance or a dedicated car. Therefore, the reduction in the number of the outpatient visits positively affected the quality of life of patients and caregivers also from an economical point of view due to the reduction of transportation costs.

**Table 4:** Comparison of scores from the OCBS questionnaire between timeframe 1 and 2, and appreciation rate for Neteka

	Baclofen 2 mg/ml	Baclofen 0.5 mg/ml
OCBS, time		
timeframe 1	41.5 [33.5–54.0]	36 [24–47]
timeframe 2	41.5 [30.5–52.5]	32 [22–42]
p*	< 0.0001	NA
OCBS, difficulty		
timeframe 1	43 [34–55]	35 [24–47]
timeframe 2	43 [30.5–53]	32 [22–42]
p*	0.0003	NA
Appreciation rate for Neteka	5 [5–5]	5 [5–5]

*Wilcoxon test; NA (not applicable) results are due to low numerosity

### Clinical efficacy

As expected, the degree of spasticity, measured as mean Ashworth score of the main muscular districts in the four limbs, did not change significantly comparing timeframes 1 and 2 (Wilcoxon test for paired samples). These data confirm no differences in clinical efficacy between the two baclofen brands.

## Discussion

Spasticity is a debilitating condition occurring in patients affected by damage in central nervous system, that impairs motor function and can cause pain, reduced range of motion, and increased muscle tone. According to recent estimates, spasticity affects up to 38% of stroke survivors, 53% of multiple sclerosis patients, and 78% of spinal cord injuries [7]. In all these conditions, in fact, impairment of alpha motor neurons results in an imbalance that elevates muscle excitability, mainly related to inadeguate action of gamma-aminobutyric acid (GABA) in facilitating muscle relaxation.

Baclofen is a GABA-agonist agent commonly applied in treatment of spasticity with a mechanism of action that involves spinal GABA-B receptors. Thus, this drug necessitates to pass through multiple physiological barriers, such as the blood-brain barrier, leading to the need of raising the oral dosage to manage severe spasticity. Consequently, undesirable side effects such as dizziness, drowsiness, nausea, and headaches can occur and complicate the clinical course of these patients [8, 9]. ITB allows drug administration directly into the cerebrospinal fluid, bypassing the blood-brain barrier and thus being effective at a lower dose, with minimal side effects since the plasmatic concentration is strongly reduced [1]. ITB is effective in reducing pain and frequency of spasms related to spasticity and in improving mobility, allowing to regain some level of independence especially in day-to-day activities [2]. The optimal baclofen dose is specific for each patient and it is usually reached through serial clinical evaluations and slight percentage dose increase until satisfactory results are achieved. Moreover, the daily dosage varies with time in the same patient, due to the need to increase the dose of the drug to maintain the antispastic effects, as outlined by Gündüz et al. [10]: in their 10-year follow-up study they observed a sharp increase (around 200%) during the first year, and then a flatter, but still increasing, for the subsequent 9 years.

Selection of patients eligible for ITB considers practical aspects beyond clinical criteria, including the ability to attend regular follow-up appointments for pump programming or refill and their related logistic needs. The refill interval varies among patients according to the medication concentration and the daily dosage. Among the patients referred to our center the average interval between one access and another is 197 days, ranging between 54 and 400 days.

In order to reduce the number of accesses, thus reducing the risk of device contamination by percutaneous puncture and, not negligibly, minimizing discomfort for patients and caregivers, we used to chose Baclofen at high concentration (2000 mcg/ml instead of less concentrated medication at 500 mcg/ml) in patients assuming a daily dosage of 110 mcg or more, thus obtaining the extension of the refill window [3]. However, a previous history of overdosing or side effects related to ITB was considered unsafe for the infusion of high concentration drug. The cut off value was obtained considering 6 months as the longest possible interval between refills, due to loss of medication potency in longer period, especially when considering non-compounded intrathecal baclofen [3, 11]. In our experience, however, many patients, especially those with a long history of spasticity, reported a significant increase in spasticity two or three months after refill, thus requesting more frequent refills.

In this perspective, the adoption of Neteka, assumed to be stable for up to six months on pump, has allowed a significant reduction in the frequency of refills for the majority of our patients. The most evident advantage over the traditional baclofen formulation provided by Bioindustria is the reduction of number of punctures. This reduction translates directly into fewer hospital visits, alleviating both the logistic and financial burden faced by patients and their families.

Given that many patients rely on private ambulances for transport, with an average cost of approximately €200 per trip (in our local specific situation), the financial implications are substantial. The reduction in refill frequency not only lowers the direct costs associated with transport but also reduces the physical and emotional strain on patients and their caregivers, particularly for those managing complex neurological conditions.

The impact on caregiver burden was quantitatively assessed using the OCBS, which showed a significant reduction in burden scores after the switch to Neteka. This suggests that the decreased frequency of hospital visits and the associated logistics had a meaningful positive effect on caregivers’ quality of life. Moreover, patient satisfaction, as evaluated through a dedicated questionnaire, remained high, with equivalent therapeutic efficacy between the two formulations. Clinical assessments using the Ashworth scale corroborated these findings, confirming that the transition to Neteka did not compromise spasticity control.

## Conclusions

Overall, the introduction of Neteka represents a valuable advancement in ITB, offering tangible benefits in terms of maintained clinical efficacy, reduced patient and caregiver burden and cost savings. These findings support a broader adoption of Neteka in clinical practice, particularly for patients requiring long-term ITB therapy. The reduction in logistic demands on caregivers, as observed with the use of Neteka, not only improves caregiver satisfaction but also enhances the overall feasibility of ITB therapy. By alleviating the burden of frequent hospital visits for pump refills, particularly in patients requiring lower dosages, the barriers to implementing ITB systems will be significantly lowered.

## Data Availability

Raw data supporting the findings of this study are available on request from the corresponding author.

## Abbreviations

GABA: Gamma-aminobutyric acid
ITB: Intrathecal baclofen therapy
OCBS: Oberst Caregiving Burden Scale
